# Evaluation of Thyroid Function Status Among Diagnosed Cases of Polycystic Ovarian Syndrome in a Tribal-Preponderant State in India

**DOI:** 10.7759/cureus.83648

**Published:** 2025-05-07

**Authors:** Asha Oroan, Pradeep Minz, Anit Kujur, Bela R Ekka

**Affiliations:** 1 Biochemistry, Medinirai Medical College and Hospital, Medininagar, IND; 2 Physiology, Rajendra Institute of Medical Sciences, Ranchi, IND; 3 Community Medicine, Rajendra Institute of Medical Sciences, Ranchi, IND; 4 Biochemistry, Rajendra Institute of Medical Sciences, Ranchi, IND

**Keywords:** hypothyroidism, menstrual disorder, polycystic ovarian syndrome, thyroid, thyroid function status

## Abstract

Introduction: Menstrual disorders and infertility are among the most common physical and psychological stressors affecting women of reproductive age. In this study, we aimed to determine the prevalence of hypothyroidism among polycystic ovarian syndrome (PCOS) patients diagnosed based on the Rotterdam criteria.

Materials and methods: A cross-sectional study was conducted among 139 cases of diagnosed PCOS at a tertiary care center in Jharkhand, India, from October 2019 to May 2021. Patients attending the Outpatient Department of Obstetrics and Gynecology and diagnosed with PCOS by radiology experts via ultrasound were enrolled in the study. Diagnosis of PCOS was confirmed by the Rotterdam criteria for PCOS.

Results: Out of 139 participants, 29 (20.87%) had thyroid-stimulating hormone levels of >5 μIU/mL, and 110 (79.13%) had thyroid-stimulating hormone (TSH) levels of <5 μIU/mL, resulting in a hypothyroidism prevalence of 20.87%. Body mass index (BMI), waist-to-hip ratio (WHR), total testosterone, serum luteinizing hormone, and thyroid-stimulating hormone levels were higher among PCOS patients than the reference values.

Conclusion: As hypothyroidism is linked to reproductive hormone imbalances, and its symptoms often overlap with PCOS, assessing thyroid function alongside reproductive hormone profiles can improve understanding and management of PCOS.

## Introduction

Menstrual disorders and infertility are among the most common physical and psychological stressors affecting women of reproductive age, with polycystic ovarian syndrome (PCOS) and hypothyroidism being the leading causes of menstrual irregularities. PCOS, a prevalent endocrine disorder among women in India, affects approximately 5%-10% of the reproductive-age population [1]; it is a heterogeneous androgen excess disorder characterized by varying degrees of reproductive and metabolic dysfunctions. Metabolic disturbances, such as insulin resistance, hyperinsulinemia, and dyslipidemia, are commonly observed in most women with PCOS. Hyperandrogenism, resulting from excessive ovarian and/or adrenal androgen secretion, can lead to clinical manifestations such as hirsutism, acne, and male-pattern baldness [2]. Both genetic and environmental factors contribute to the etiology of PCOS.

PCOS can have significant long-term consequences, including an increased risk of developing endometrial hyperplasia or neoplasia. The Rotterdam Consensus of 2003, established by the European Society of Human Reproduction and Embryology (ESHRE) and the American Society for Reproductive Medicine (ASRM), remains the most widely used diagnostic criteria for PCOS in both clinical and research settings [3]. According to this consensus, PCOS is diagnosed when at least two of the following three features are present: hyperandrogenism (clinical or biochemical), ovulatory dysfunction (often leading to menstrual irregularities), and polycystic ovarian morphology detected via ultrasound. Diagnosis is confirmed only after ruling out other causes of anovulation or androgen excess, such as hypogonadism, thyroid disorders, hyperprolactinemia, 21-hydroxylase deficiency, Cushing's syndrome, and androgen-producing tumors.

Hypothyroidism, another prevalent endocrine disorder in women, often coexists with PCOS and contributes to menstrual irregularities and infertility. In reproductive-age women, the prevalence of hypothyroidism is estimated to be 2%-4%, making it a significant factor in infertility [4,5]. Thyroid hormone disorders and thyroid autoimmunity have been linked to an increased risk of infertility, spontaneous miscarriage, preterm delivery, and metabolic dysfunctions, many of which are also commonly observed in PCOS. Studies suggest a higher prevalence of subclinical hypothyroidism and thyroid autoimmunity in women with PCOS compared to the general female population [6-8]. Emerging research continues to explore the association between thyroid function, thyroid autoimmunity, and metabolic parameters in PCOS, particularly concerning dyslipidemia and insulin resistance.

Studies have shown that hypothyroidism may affect the clinical presentation of PCOS in multiple systems, which also compromises metabolism and immunity, elevating the risk of infertility and irregularities in menstrual cycles among women of reproductive age [9]. Given the significant overlap in symptoms between PCOS and hypothyroidism (e.g., obesity, menstrual disturbances due to anovulation, acne, hirsutism, infertility, miscarriages, and insulin resistance), assessing thyroid function is crucial in all women presenting with menstrual irregularities and infertility.

Thyroid hormone deficiency has profound effects on various organ systems, including the reproductive system. Chronic hypothyroidism can disrupt gonadotropin secretion by raising serum prolactin levels, leading to menstrual cycle irregularities and impaired fertility due to abnormalities in the luteal phase or anovulation [10,11]. Although the precise relationship between PCOS and thyroid disorders remains unclear, the frequent co-occurrence of both conditions creates a diagnostic challenge. Many cases of hypothyroidism are subclinical and first detected during the evaluation of PCOS. Hormonal disturbances associated with hypothyroidism further complicate the clinical picture.

As PCOS and hypothyroidism share overlapping symptoms and are often interrelated, evaluating thyroid function alongside reproductive hormone profiles may improve the understanding of their etiology and aid in more effective management. As research continues to explore the connection between thyroid function and PCOS, better diagnostic and therapeutic approaches may emerge. Therefore, in the present study, we aimed to determine the prevalence of hypothyroidism and explore the relationship between thyroid function status and clinical parameters (such as body mass index (BMI), waist-to-hip ratio (WHR), and testosterone levels) among PCOS patients based on the Rotterdam criteria.

## Materials and methods

The present study was conducted at a tertiary care center in Jharkhand, India, from October 2019 to May 2021. A total of 139 cases were reviewed after receiving ethical approval from the relevant institutional ethical committee (approval number 215, date: December 21, 2019). Patients aged 15-45 years attending the Obstetrics and Gynecology Outpatient Department of a tertiary care center in Jharkhand and diagnosed with PCOS through ultrasound by the Radiology Department were enrolled in the study. Subjects with other causes of hyperandrogenism (e.g., congenital adrenal hyperplasia, virilizing tumor, hyper-prolactinoma, and Cushing's syndrome) and hypogonadism were excluded. In addition, patients receiving treatment for hypothyroidism or hyperthyroidism and who were using oral contraceptives were also excluded from the study. Subjects were finally diagnosed by the ESHRE/ASRM-endorsed Rotterdam PCOS criteria.

Before starting the study, each subject was informed about the purpose of the study, and informal informed consent was obtained. Data were collected through interviews based on questionnaires. Anthropometric parameters were measured, and blood samples were collected for each patient, with precautions to prevent hemolysis and contamination. Further processing and biochemical analysis of blood samples were done with a fully automated chemistry autoanalyzer (CS-T240, Dirui, Changchun, China) and chemiluminescence testing (Architect Plus i1000 SR, Abbott, Abbott Park, IL).

The interview comprised two sections. The first section was related to demographic variables and family history of diseases such as PCOS, hypertension, diabetes, and obesity. The second section contained items related to nutritional habits, type of diet, anthropometric measurements, and any addictions (e.g., chewing tobacco, smoking, and drinking alcohol). The interview was followed by collection of blood samples for biochemical assessment.

Collection of blood samples

All patients were asked not to eat any fatty foods on the night before blood testing. The following precautions were followed while drawing blood samples: hand washing and wearing of gloves; changing of gloves after each patient; disposal of waste as per biomedical waste management rules; destroying of needles with a hub cutter immediately after drawing each sample; cleaning of blood spills with disinfectant (freshly made 10% bleach); and placing blood collection equipment away from patients, especially children.

A sterile, dried vial was used to collect the sample, which was allowed to clot for estimation of serum hormones. A portion of the drawn blood was placed in a fluoride vial containing sodium fluoride and potassium oxalate to estimate blood glucose. Needles of 20 or 22 G size were used for drawing blood samples and were disposed of per biomedical waste rules. A cotton swab with 70% isopropyl alcohol was used to clean, wipe, and sterilize the skin prior to collection.

Blood collection procedure

For the collection of blood samples, the antecubital vein was preferred. First, the surface of the skin was palpated meticulously, and the direction of the veins was navigated with the index finger. Thrombosed veins (i.e., those feeling like a cord and rolling easily) were avoided, as they lack the ability to recover quickly. In cases where superficial veins were not readily apparent, blood was forced into the vein by massaging the arm from the wrist to the elbow; then, the site was tapped with the index and second finger, or the extremity was lowered to allow the vein to fill. Once the preferred site was selected, gloves were donned, and the venipuncture site was cleansed using an alcohol preparation, following a circular motion starting at the site and moving outward. The site was allowed to dry before proceeding. A tourniquet was applied 3-4 inches above the puncture site while ensuring that it was not too tight. The venipuncture was performed with the patient's arm in a comfortable position. The tourniquet was promptly removed once blood appeared in the syringe.

Approximately 5-6 mL of blood was withdrawn. After collection, a cotton ball or gauze was placed over the site, and the needle was removed smoothly and cautiously to prevent bruising. Gentle pressure was applied to the puncture site, and the patient was instructed to maintain pressure for 3-5 minutes to prevent oozing. The collected blood samples were then transferred into appropriate vials.

For plasma samples, blood was collected in a fluoride vial, shaken, and placed in a rack at room temperature before being centrifuged at 3000 rpm for 10 minutes. For serum samples, the blood was allowed to clot at room temperature for at least 30 minutes before centrifugation at 3000 rpm for 10 minutes, within two hours of collection. Serum and plasma samples were analyzed on the same day using a fully automated autoanalyzer (CS-T240, Dirui) and a chemiluminescence analyzer (Architect Plus i1000 SR, Abbott).

Data were collected and entered into a Microsoft Excel (version 13) spreadsheet (Microsoft Corp., Redmond, WA) and analyzed using the Statistical Package for the Social Sciences (SPSS) (version 26) (IBM Corp., Armonk, NY). Mean and standard deviation were calculated for comparison, with reference levels and Student's unpaired t-test used to find any statistical association between parameters. p-values of less than 0.05 were considered statistically significant.

## Results

The present study included 139 participants diagnosed with PCOS. A significant portion were over the age of 20 (118 (84.90%)) and were dwelling in rural areas (80 (57.55%)). Most of the participants with PCOS belong to the Hindu religion by faith (76, 54.67%) and were non-tribal (106 (76.26%)). In terms of educational attainment, 78 (56.20%) had completed their education at the middle school level. Regarding family history among PCOS participants, 39 (28%) had a history of PCOS, 14 (10%) had a history of hypertension, 39 (28.10%) had a history of diabetes, and 38 (27.30%) had a history of obesity (Table 1).

**Table 1:** Demographics and family history of the participants with PCOS *Local religion of Jharkhand PCOS: polycystic ovarian syndrome

Variables		Frequency (N = 139)	Percentage
Age	<20 years	21	15.10%
	>20 years	118	84.90%
Place of residence	Rural	80	57.55%
	Urban	59	42.45%
Religion	Hindu	76	54.67%
	Muslim	20	14.38%
	Christian	13	9.36%
	Sarna*	30	21.59%
Ethnicity	Tribal	33	23.74%
	Non-tribal	106	76.26%
Educational status	Illiterate	14	10.00%
	Primary	39	28.00%
	Middle	78	56.20%
	Matriculation	5	3.60%
	Intermediate and above	3	2.20%
Family history of PCOS	Present	39	28.00%
	Absent	100	72.00%
Family history of hypertension	Present	14	10.00%
	Absent	125	90.00%
Family history of diabetes	Present	39	28.10%
	Absent	100	71.90%
Family history of obesity	Present	38	27.30%
	Absent	101	72.70%

In the current study, over half of the participants (76, 54.70%) reported following a vegetarian diet, while 63 (45.30%) participants adhered to a non-vegetarian diet. A significant portion of the participants (80 (57.56%)) consumed junk food three or more times a week. Among the 139 women with PCOS, 40 (28.77%) were classified as obese, and 59 (42.44%) were found as overweight. Additionally, 47 (33.81%) participants had a history of some form of addiction (Table 2).

**Table 2:** Nutritional habits, anthropometrics, and presence of any addiction BMI: body mass index

Variables		Frequency (N = 139)	Percentage
Type of diet	Vegetarian	76	54.70%
	Non-vegetarian	63	45.30%
Consumption of junk foods	≤3 days a week	59	42.44%
	≥3 days a week	80	57.56%
BMI	Underweight (<18.5 kg/m²)	2	1.43%
	Normal (18.5-24.9 kg/m²)	38	27.33%
	Overweight (25-29.9 kg/m²)	59	42.44%
	Obese (>30 kg/m²)	40	28.77%
History of any addiction (tobacco in any form and alcohol)	Yes	47	33.81%
	No	92	66.19%

The mean age of the study sample was 24.64 ± 4.23 years, and the mean body mass index (BMI) was 27.49 ± 3.35, which was more than the upper limit of the normal BMI level, as seen in Table 3. The mean waist-to-hip ratio (WHR) was 0.86 ± 0.034, which was also found to be above the normal reference level (<0.85). The mean serum fasting insulin and serum testosterone levels were found to be 12.75 ± 8.71 μU/mL and 50.27 ± 21.16 ng/dL, respectively, which are within the normal cutoff level. The mean luteinizing hormone and follicle-stimulating hormone levels were found to be 15.16 ± 8.19 mIU/mL and 6.88 ± 3.15 mIU/mL, respectively, which are also above the normal reference value mentioned in Table 1. The mean prolactin level (14.76 ± 7.04 ng/mL) was within the normal reference range. The mean thyroid-stimulating hormone (TSH) level was 4.08 ± 3.14 μIU/mL, with a standard deviation suggesting that some participants had TSH levels outside the normal range of 0.35-4.94 μIU/mL. Free T3 and T4 levels were found within the normal limit with values of 2.07 ± 0.36 pg/mL and 1.08 ± 0.23 ng/dL, respectively.

**Table 3:** Mean parameters of the participants with PCOS PCOS: polycystic ovarian syndrome

Parameter	Mean ± standard deviation	Reference value
Age (years)	24.64 ± 4.23	15-45
Body mass index (kg/m^2^)	27.49 ± 3.35	18.5-24.9
Waist-to-hip ratio	0.86 ± 0.034	<0.85
Fasting blood sugar (mg/dL)	84 ± 14.98	70-110
Serum fasting insulin (μU/mL)	12.75 ± 8.71	2.6-24
Serum testosterone (ng/dL)	50.27 ± 21.16	6-86
Luteinizing hormone level (mIU/mL)	15.16 ± 8.19	1.80-11.78
Follicle-stimulating hormone level (mIU/mL)	6.88 ± 3.15	3.03-8.08
Prolactin level (ng/mL)	14.76 ± 7.04	5.18-26.53
Thyroid-stimulating hormone level (μIU/mL)	4.08 ± 3.14	0.35-4.94
Free T3 (pg/mL)	2.07 ± 0.36	1.71-3.71
Free T4 (ng/dL)	1.08 ± 0.23	0.70-1.48

Table 4 shows the thyroid function status of the 139 participants. Out of 139 participants, 29 (20.87%) were found suffering from hypothyroidism, with TSH levels of >5 μIU/mL, whereas the majority of the participants (110 (79.13%)) had normal thyroid function test results, with TSH levels within the normal limit of less than 5 μIU/mL. The prevalence of hypothyroidism was found to be 20.87%.

**Table 4:** Thyroid function status according to TSH level (cut-off: 5 μIU/mL) among study population (N = 139) TSH: thyroid-stimulating hormone

Thyroid function status	Frequency (N = 139)	Percentage
Hypothyroidism (>5 μIU/mL)	29	20.87%
Euthyroidism (<5 μIU/mL)	110	79.13%

Table 5 shows the comparison of parameters between euthyroid and hypothyroid PCOS participants. The mean BMI was 30.67 ± 2.53 among PCOS participants suffering from hypothyroidism and 26.79 ± 3.10 among PCOS participants who were euthyroid, and the difference shows that BMI was higher among PCOS participants who were suffering from hypothyroidism. WHR was also found to be higher among the hypothyroid participants when compared to euthyroid participants who had normal WHR, and this difference was statistically significant. Finally, serum testosterone level was also higher among hypothyroid participants (64.60 ± 22.34 ng/dL) as compared to euthyroid participants (47.13 ± 19.64 ng/dL), and this difference was statistically significant.

**Table 5:** Comparison of clinical parameters among euthyroid and hypothyroid patients with PCOS (N = 139) *Statistical significance PCOS: polycystic ovarian syndrome

Parameter	Euthyroid PCOS (n = 110)	Hypothyroid PCOS (n = 39)	t-test value	p-value
Body mass index	26.79 ± 3.10	30.67 ± 2.53	7.0016	0.0001*
Waist-to-hip ratio	0.86 ± 0.03	0.89 ± 0.02	5.7983	0.0001*
Fasting insulin (μU/mL)	12.83 ± 8.65	12.42 ± 9.16	0.2504	0.8026
Serum testosterone (ng/dL)	47.13 ± 19.64	64.60 ± 22.34	4.6014	0.005*

## Discussion

As the incidence of infertility and its associated health complications has increased, identifying and addressing the root cause has become increasingly important. Among women, menstrual irregularities are the leading cause of infertility, apart from anatomical and genetic factors. The primary contributors to irregular menstruation include ovarian conditions such as PCOS, as well as systemic disorders such as hypothyroidism, hyperprolactinemia, and hyperinsulinemia.

PCOS and hypothyroidism are the most prevalent endocrine disorders among women of reproductive age. In clinical practice, although some patients respond well to PCOS treatment with restored menstruation and ovulation, others do not show improvement. Further evaluation of these non-responding individuals often reveals undiagnosed or untreated hypothyroidism, typically in a subclinical or overt state. Screening studies play a crucial role in assessing the prevalence of hypothyroidism and understanding its epidemiological impact. However, research comparing thyroid function in PCOS patients remains limited across various regions of India. Against this backdrop, we conducted a study to evaluate thyroid function in newly diagnosed PCOS patients. This cross-sectional study involved 139 women diagnosed with PCOS based on the revised Rotterdam criteria (2003) and attending the Outpatient Department of Obstetrics and Gynecology for menstrual irregularities and infertility.

Younger patients (under 20 years old) were notably fewer in number among those diagnosed with PCOS compared to participants over 20 years of age, particularly in rural areas (Table 1). This observation aligns with the findings of Ganni et al. [12] but contrasts with the research conducted by Vidya Bharathi et al. [13], who indicated that women living in urban areas have a higher likelihood of developing PCOS. This discrepancy may be attributed to differences in lifestyle and dietary habits. Additionally, Ganni et al. reported that a significant portion of the subjects identified as Hindu and were non-tribal [12], which is consistent with the observations made in our current study. The findings of our study regarding educational status also mirrored the evidence presented by Ganni et al. [12]. In our current research on family history in women with PCOS, we observed a comparable prevalence of PCOS, diabetes, and obesity. This finding aligns with the study conducted by Moini et al. [14]. In relation to the history of addiction, a notable portion of PCOS participants reported no history of addiction, which contrasts with the findings from a study done by Pau et al. [15], indicating that smoking could exacerbate the already elevated risk of metabolic syndrome in women with PCOS.

In the present study, over half of the participants with PCOS adhered to a vegetarian diet (Table 2), which aligns with the results of the study by Ganie et al. [16]. Their findings indicated that women with PCOS who followed an Indian vegetarian diet exhibited elevated levels of pro-inflammatory markers and reduced levels of anti-inflammatory markers. A considerable number of participants who consumed junk food three or more times a week demonstrated a notable link between diet and the development of PCOS, a finding that aligns with the research conducted by Xenou et al. [17].

Considering a TSH level of 5 μIU/mL as the cutoff value to diagnose hypothyroidism, 29 (20.87%) participants had a TSH level of >5 μIU/mL, and 110 (79.13%) participants had a TSH level of <5 μIU/mL (Table 1). Therefore, hypothyroidism had a prevalence of 20.87% among the 139 newly diagnosed women of reproductive age with PCOS. In contrast, 110 (79.13%) individuals were euthyroid. A study by Sinha et al. reported similar findings [18], where thyroid disorders were identified in 22 out of 80 PCOS patients (27.5%), as compared to nine individuals in the control group (11.25%) (p < 0.05). In addition, subclinical hypothyroidism was present in 18 patients (22.5%) (8.75% in the control group), whereas two (2.5%) patients had overt hypothyroidism.

Likewise, in a study of 196 PCOS patients by Nanda et al., 26 (13.26%) had hypothyroidism, two (1.02%) had hyperthyroidism, and 168 (85.71%) had normal thyroid function [19]. Among the 26 hypothyroid cases, 15 (57.69%) had subclinical hypothyroidism and 11 (42.30%) had overt hypothyroidism. A study by Ramanand et al. also reported a hypothyroidism prevalence of 15.9% among PCOS patients [20], aligning closely with our study. Similarly, Ganvir et al. found subclinical hypothyroidism in 16 (26.6%) cases and overt hypothyroidism in 12 (20%) cases [21], comparable to our findings.

In our study, 42.44% of PCOS patients were overweight, and 28.77% were obese. As seen in Table 3, the mean BMI was lower in euthyroid individuals (26.79 ± 3.10) than in hypothyroid individuals (30.67 ± 2.53), and this difference was statistically significant (p = 0.0001). Obesity is a common feature of PCOS, often contributing to hypothyroidism. A similar finding was documented by Lim et al., who reported a positive association between obesity and PCOS [22]. This link may be due to increased pro-inflammatory conditions and insulin resistance in obesity, which could reduce deiodinase-2 activity at the pituitary level, leading to relatively lower T3 and higher TSH levels [23].

Among our study sample, 69.79% of PCOS patients had a WHR of >0.85, and 30.21% had a WHR of <0.85. The mean WHR was 0.863 ± 0.033 in euthyroid patients and 0.896 ± 0.022 in hypothyroid patients, and this difference was statistically significant (p = 0.001). Similar findings were reported by Zhang et al. [24], where WHR was significantly higher in PCOS patients than in the control group, suggesting an association between increased WHR and hypothyroidism.

Regarding testosterone levels, 87% of PCOS patients had total serum testosterone below 86 ng/dL, and 12.97% had levels above this threshold. Testosterone levels were significantly higher in hypothyroid patients than in the euthyroid group. The mean testosterone level was 47.136 ± 19.642 in euthyroid patients and 64.606 ± 22.34 in hypothyroid patients, and this difference was statistically significant (p = 0.005). Similar results were observed in the study by Zhang et al. [24], where testosterone levels were elevated in hypothyroidism patients as compared to the control group; this suggests that increased TSH levels correlate with higher testosterone, indicating that hypothyroidism contributes to hyperandrogenism and its associated effects, such as PCOS. The coexistence of hypothyroidism may exacerbate hyperandrogenic symptoms, potentially leading to persistent menstrual irregularities, infertility, and ultrasound-detected ovarian cysts in patients receiving PCOS treatment. These findings highlight the importance of assessing serum TSH levels in PCOS patients.

This study did have some limitations. Participants were selected from a single center, without representing diverse backgrounds, reducing the generalizability of the results. Furthermore, as this was a single-center study, the sample size was relatively small due to time constraints, which limited the statistical power of the study.

## Conclusions

Ultimately, the findings suggest that many PCOS patients have undiagnosed hypothyroidism, which exacerbates hyperandrogenism. The significant difference in serum testosterone levels between the hypothyroid and euthyroid groups reinforces the need for a comprehensive approach to managing PCOS, taking into consideration not just reproductive hormones but also thyroid function. Given that hypothyroidism can influence insulin resistance, fertility outcomes, and the overall quality of life for PCOS patients, it is essential for clinicians to monitor thyroid function as part of the broader management of the syndrome. Addressing thyroid abnormalities in these patients may improve both metabolic and reproductive outcomes, highlighting the need for individualized treatment strategies. Early diagnosis and appropriate investigations can help prevent disease progression and associated complications. Further research is essential to better understand the mechanisms linking hypothyroidism and PCOS, as well as to evaluate the impact of thyroid hormone treatment on the clinical management of PCOS patients.
